# IMPACT OF ELDER ABUSE ON SUBSEQUENT COGNITIVE DETERIORATION IN OLDER ADULTS WITH COGNITIVE IMPAIRMENT

**DOI:** 10.1093/geroni/igz038.1059

**Published:** 2019-11-08

**Authors:** Boye FANG, Elsie Yan

**Affiliations:** 1 Hong Kong Polytechnic University, Hong Kong Polytechnic University, Hong Kong; 2 The Hong Kong Polytechnic University, Hong Kong, Hong Kong

## Abstract

Objectives: Considering the rapidly increasing older population, elder abuse has become a major public health problem. Using longitudinal data, this study examines the impact of elder abuse on subsequent cognitive deterioration. Methods: At baseline, one-thousand-and-two older adults (aged≥55 years) with a clinically valid diagnosis of mild-to-moderate cognitive impairment were consecutively recruited from the geriatric and neurological departments of three Grade-A hospitals in Guangdong Province of People’s Republic of China from 2015 to 2016 and 958 of them have completed the present follow-up study after two years. The major independent variables were psychological abuse, physical abuse, caregiver neglect, and financial exploitation experienced at baseline. The outcome variable was cognitive deterioration defined by repeated measures using the Mini-Mental State Examination. Covariates included demographic characteristics, neuropsychiatric symptoms, depression, and IADL impairment. Results: According to the mixed-effect model, elder abuse at baseline did not have significant association with baseline cognitive impairment. However, experience of physical abuse (coefficient 0.11; 95% CI 0.06-0.15), psychological abuse (coefficient 0.28; 95% CI 0.09-0.47), and caregiver neglect (coefficient 0.03; 95% CI 0.01-0.05) at baseline predicted greater cognitive deterioration over the two-year observation period. Other contributing factors for greater cognitive deterioration included neuropsychiatric symptoms and depression. Discussion: Although no significant cross-sectional association between elder abuse and cognitive impairment was observed, physical abuse, psychological abuse, and caregiver neglect at baseline was found to have a long-term prominent effect on subsequent cognitive deterioration over the two-year observation period. The present results su

